# Delayed diagnosis of myocardial infarction in a young man with a blunt chest trauma

**DOI:** 10.15171/jcvtr.2019.42

**Published:** 2019-07-21

**Authors:** Marzieh Nikparvar, Sayed Mohammadbagher Asghari, Hossein Farshidi

**Affiliations:** ^1^Cardiovascular Research Center, Hormozgan University of Medical Sciences, Bandar Abbas, Iran; ^2^Shaheed Mohammadi Educational Hospital, Bandar Abbas, Iran

**Keywords:** Myocardial Infarction, Blunt Chest Trauma, Percutaneous Coronary Intervention

## Abstract

A 19 year old man was admitted to emergency department (ED) because of motorcycle to motorcycle accident. In ED he was evaluated as a multiple trauma patient and after overnight observation, was discharged with stable vital signs (VS) next morning. Twenty four hours later, he developed chest pain and dyspnea, and was admitted to ED for the second time. This time he was evaluated as a chest trauma patient and chest X ray and CT scanning were performed. According to cardiology consult, an electrocardiogram was recorded and extensive anterolateral ST elevation myocardial infarction (STEMI) was confirmed. Coronary angiography revealed total thrombotic occlusion of left anterior descending artery (LAD) from ostium. Percutaneous coronary intervention (PCI) was performed and LAD stented successfully. After 10 days of ICU admission, he was discharged with an ejection fraction (EF) of 35%.

## Introduction

Acute coronary occlusion after blunt chest trauma (BCT) is one of the disastrous conditions which many physicians could face it. Although it has been reported rarely^1,2^ patients suffering from BCT may have cardiac or pulmonary contusion, muscular injuries or rib fractures.

Myocardial injury, if not diagnosed promptly and on time^3,4^ or treated with delay, could lead to worse outcomes and increase the morbidity and mortality. Several studies and case reports of chest trauma other than car accident have been reported^5-7^ which have resulted in myocardial infarction (MI). In this case a young man with BCT, a victim of motorcycle to motorcycle accident is presented who suffered from resultant MI and was treated with PCI and drug eluting stent (DES).There is a time delay in diagnosis of MI that can be life threatening in a young patient with such a history.

## Case Report

A 19 years old college student was transported to a referral hospital in Bandar Abbas-IRAN by EMS 30 minutes after motorcycle to motorcycle accident. In this accident the patient was thrown away from the motorcycle, facing the guard rail of the road which caused a trauma to chest, neck and extremities.

He was evaluated as a case of multiple trauma and was admitted in surgery ward.

Patient had been examined by emergency specialist, orthopedic surgeon and neurosurgeon.

His cervical CT scan showed no fractures or dislocations or displacements. He was discharged after overnight observation with stable general condition. Twenty four hours after his first admission he was re-admitted to ED with severe chest pain and dyspnea. At first examination in ED blood pressure was 105/80 mm Hg, pulse rate was 100/min ,respiratory rate was 30/min,O2 saturation was 96% in room air, auscultation revealed muffled heart sounds and diffuse crackles in both lungs. He was suspected of chest injury or lung contusion and was visited by a thoracic surgeon.

**Figure 1:**
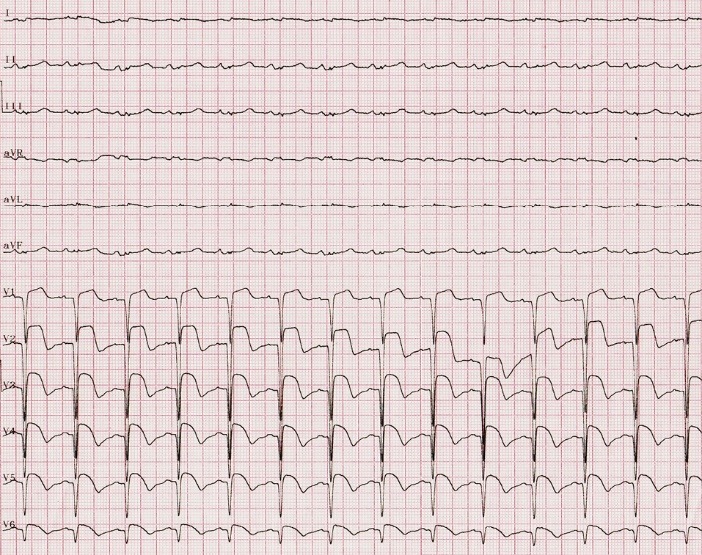
First electrocardiography shows ST-segment elevation and Q waves in leads V1 to V6.

**Figure 2:**
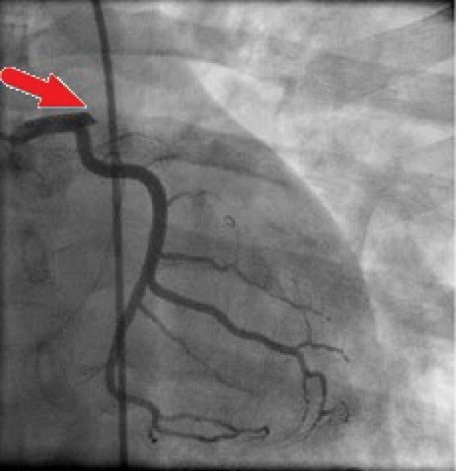
Coronary angiography in RAO projection showing total occlusion of LAD.

His chest CT scan showed bilateral pulmonary consolidation and infiltration, for which antibiotics were started along with conservative therapy. His ECG revealed “ST elevation” in anterolateral leads (I, aVL, V1-V6) (Figure 1). In Echocardiography left ventricular ejection fraction (LVEF) was 25%-30% with regional wall motion abnormality in anteroseptal and apical segments. Troponin was positive and CK-MB was 547 U/L. Patient was premedicated with 600 mg Clopidogrel, 325 mg ASA, and 5 μL/min serum TNG, 2 μL/kg infusion of Eptifibatide, 40 mg intravenous furosemide and 80 mg Atorvastatin. Performed angiography showed totally occluded LAD with large thrombus from ostial part (Figure 2). RCA and LCX arteries were normal. A large amount of clot was extracted by performing thrombosuction that resulted in restoring LAD flow. Finally, LAD was stented with 3.5*20 mm resolute integrity stent (DES). After revascularization, TIMI flow 3 was recovered.

During procedure he developed several episodes of ventricular tachycardia (VT) and ventricular fibrillation (VF) that were successfully managed with DC shock and intravenous Amiodarone. During resuscitation and advanced cardiac life support, the patient was intubated and transferred to intensive care unit (ICU) with a BP of 90/60 mm Hg, PR of 110/min, intubated and ventilated by transbag respiratory support. On the following day patient was stable with no arrhythmia, and the echocardiography showed no pericardial effusion with LVEF of approximately 25%-30%. On the third day he was extubated and transferred to coronary care unit (CCU). After a week of treatment with ASA, clopidogrel, captopril, carvedilol, furosemide and enoxaparin he was discharged with ASA80 mg/d, Captopril 25 TDS, atorvastatin 40 mg/d, spironolactone 25 mg/d, furesemide tab. 20 mg twice daily, pantoprazole 20 mg/d clopidogrel 75 mg daily, and carvedilol 6.25 twice daily. After one year of follow up his EF was improved to 35% with good functional capacity (FC I-II).

## Discussion

Blunt cardiac trauma is used instead of cardiac contusion in literature. In this condition, according to Mann et al injury is caused either by direct transfer of energy to heart or compression of heart between sternum and vertebral column during the accident or at time of cardiac resuscitation.^8^

Acute MI following BCT is a rare condition. Several reports of acute MI after BCT from different countries have been published.^9-11^ The most of the victims are young males under 45 years old with no risk factors and LAD coronary artery is the most involved vessel in most of the reports. This is probably a result of LAD’s anterior position^1^. As we know the most important coronary artery is the LAD that supplies more than 60% of myocardium and lack of collaterals in young patients worsens the prognosis. The time is the most important factor influencing post MI prognosis. As early diagnosis of MI could result in early treatment preserving more myocardium which eventually results in good LVEF and better functional capacity in future. This maybe more important in younger patients because they are rarely suspected of MI and are more under diagnosed which leads to mismanagement and high morbidity and mortality. Probable mechanisms in this condition include: Intimal tear, coronary dissection, plaque rupture, coronary spasm, intraluminal thrombosis, platelet aggregation, cardiac contusion and vessel rupture.^1,12^

It is important to consider acute MI in patients with BCT that complain of chest pain,

tachypenea, and hypotension, hence ECG, echocardiography, measurement of cardiac enzymes are necessary. Time delay in receiving appropriate therapy in case of MI is the most important factor for mortality and morbidity.^13,14^ Most of the case reports are in favor of LAD involvement in young men just as our case.^1,15,16^

Our case is very young comparing with reported cases and had no familial or traditional risk factors. Most of the reports are about car accident but there are several reports describing other kind of trauma, like bicycle or sports events,^5,17^ In his first admission to ED, cardiac problems were not been suspected as the patient was a young man with no known cardiac risk factors victim of trauma to chest, neck and extremities (as it has been reported in lot of cases), thus treatment for MI was initiated late.^18^ Angiography is the diagnostic procedure of choice for such condition providing the anatomy of the involved vessels which helps to choose the best therapeutic plan, PCI is recommended therapy and is choice especially in patients with multiple trauma with high chance of bleeding in other sites. Although several cases showed spontaneous thrombolysis and self-limitation of infarct size in the involved artery most patients are scheduled for PCI or CABG.^19^ Coronary CT angiography is another diagnostic tool that been used and provides data about anatomy and effects of any planned procedures.^7^

Despite the high rate of vehicle accidents in Iran which mainly sufferers are the young

population, few cases of MI after BCT been diagnosed and reported^18-20^ in another words it seems that some of cases are under-diagnosed and treated.

## Conclusion

Cardiac involvement may be missed in patients with BCT. ECG should be a routine paraclinical test in the evaluation of such patients regardless of the age.

## Competing interests

All authors declare no competing financial interests exist.

## Ethical approval

An informed consent was taken from the patient for publishing this case report.
